# Supplementary material for: Structural insight into mitochondrial β-barrel outer membrane protein biogenesis
Source: Nat Commun. 2020 Jul 3;11:3290. doi: 10.1038/s41467-020-17144-1 (PMC7335169; doi:10.1038/s41467-020-17144-1)
Supplement: Supplementary file 1 — Supplementary Information [file 41467_2020_17144_MOESM1_ESM.pdf]

Supplementary Information

Structural Insight into Mitochondrial β-Barrel Outer Membrane Protein Biogenesis

Diederichs *et al.*

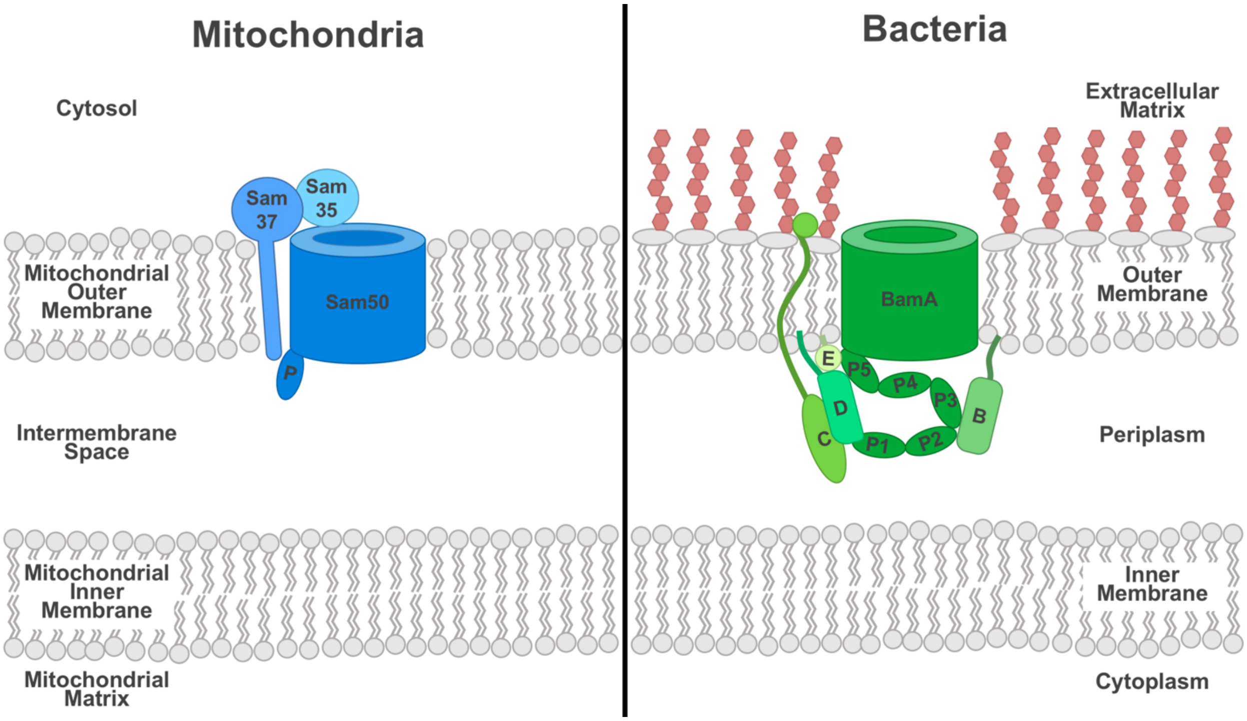

Supplementary Figure 1. Cartoon comparison of mitochondrial SAM complex and bacterial BAM complex. The core β-barrels of SAM and BAM complexes (Sam50 and BamA, respectively) span the outer membrane. Sam35 and Sam37 associate with Sam50 on the cytosolic side of the membrane while BamB, C, D, and E lipoproteins associate with BamA in the periplasm. P= polypeptide-transport-associated (POTRA) domain.

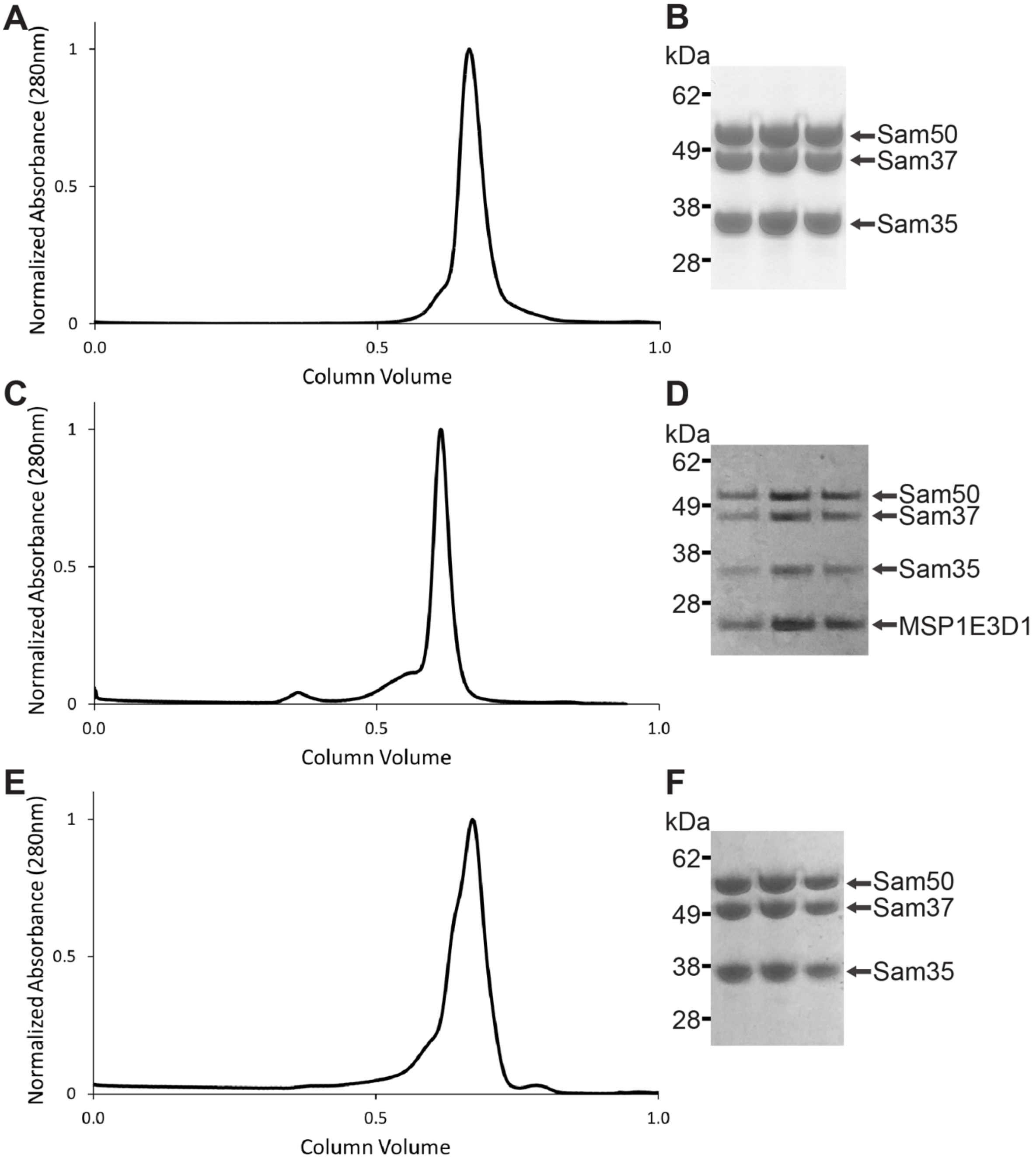

Supplementary Figure 2. Purification of the SAM complex and incorporation into lipid nanodiscs. A. Size exclusion chromatogram of the SAM complex in 0.02% LMNG, Superose 6 16/600 (GE Healthcare). **B.** SDS-PAGE of peak fractions from size exclusion chromatography in LMNG. **C.** Size exclusion chromatogram of the SAM complex incorporated into MSP1E3D1 lipid nanodiscs, Superose 6 10/300 (GE Healthcare). **D.** SDS-PAGE of peak fractions from the size exclusion of the SAM complex in lipid nanodiscs. **E.** Size exclusion chromatogram of the SAM complex exchanged into 0.02% GDN over Superose 6 16/600 (GE Healthcare). **F.** SDS-PAGE of peak fractions from size exclusion chromatography in GDN. The experiments in **B**, **D**, and **F** were repeated at least three separate times with similar results. Source data for **B**, **D**, and **F** are provided as a Source Data file.

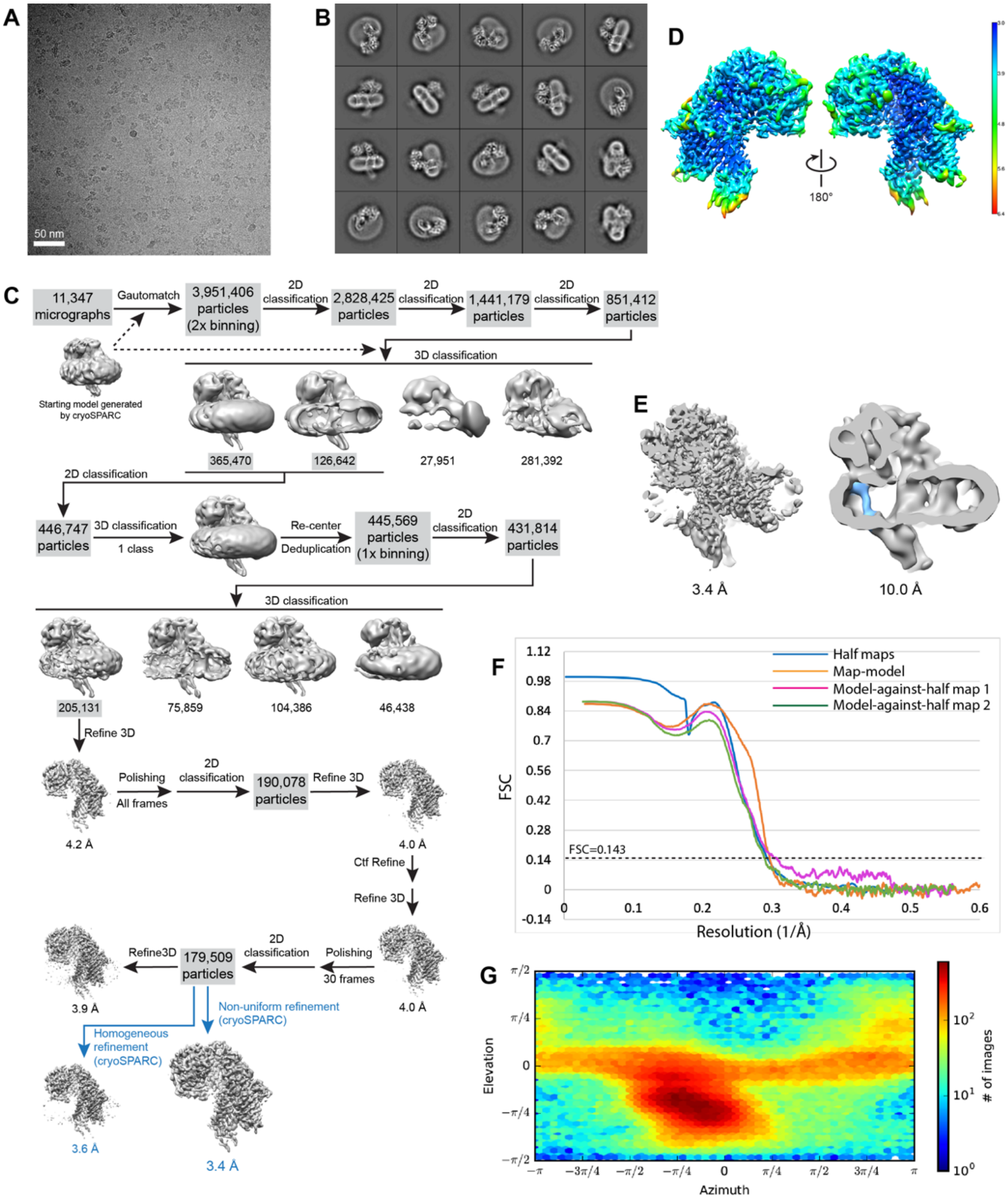

Supplementary Figure 3. CryoEM data processing of the SAM complex in lipid nanodiscs. **A.** Representative cryoEM micrograph of the SAM complex in lipid nanodiscs, selected from 11,347 micrographs. **B.** Representative 2D class averages of the SAM complex. Side length of each image box is 204 Å. 2D classification was performed 7 times during data processing and these 2D class averages were observed in all 2D classification results. **C.** Schematic diagram of cryoEM data processing procedures for the SAM complex in lipid nanodiscs. **D.** Local resolution map calculated by cryoSPARC2. **E.** The first of two predicted transmembrane α-helices in Sam37 is visible (colored in blue) when the cryoEM density map is low-pass filtered to 10 Å. **F.** Fourier Shell Coefficient (FSC) curves. **G.** Orientation distribution plot.

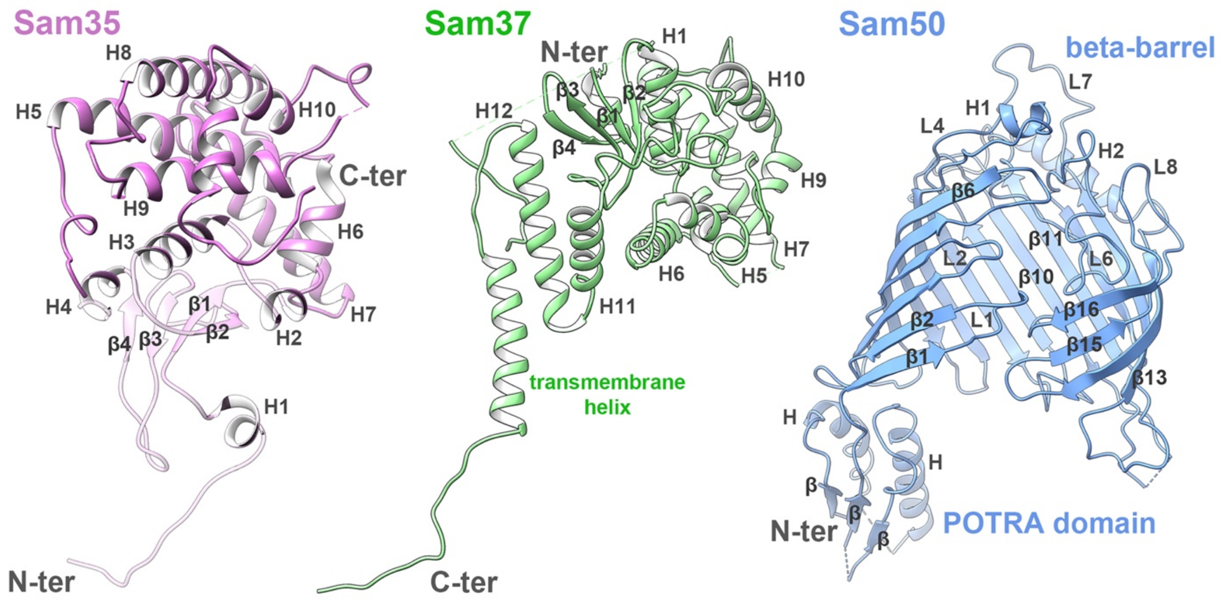

Supplementary Figure 4. Structural elements of SAM complex subunits. Sam35 in orchid, Sam37 in light green, and Sam50 in blue. H= alpha helix, β= beta-strand, L= loop.

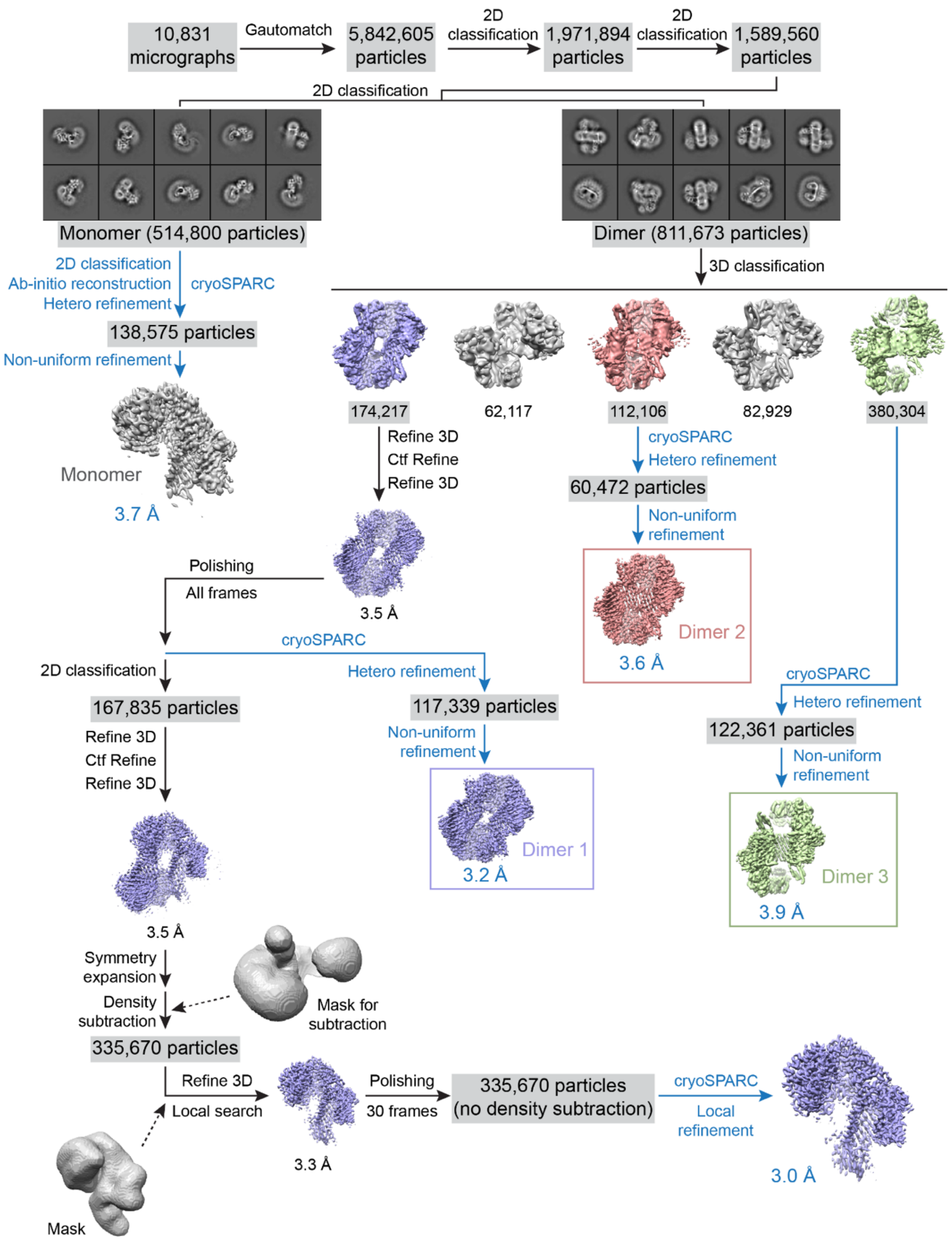

Supplementary Figure 5. CryoEM data processing of the SAM complex in detergent GDN. The procedures carried out using different software packages are depicted in black for RELION3 and blue for cryoSPARC2. The particles of monomer and dimer were sorted by 2D classification. Side length of each image box of the 2D class averages is 220 Å. 2D classification was performed more than 3 times and these representative 2D class averages were observed repeatedly.

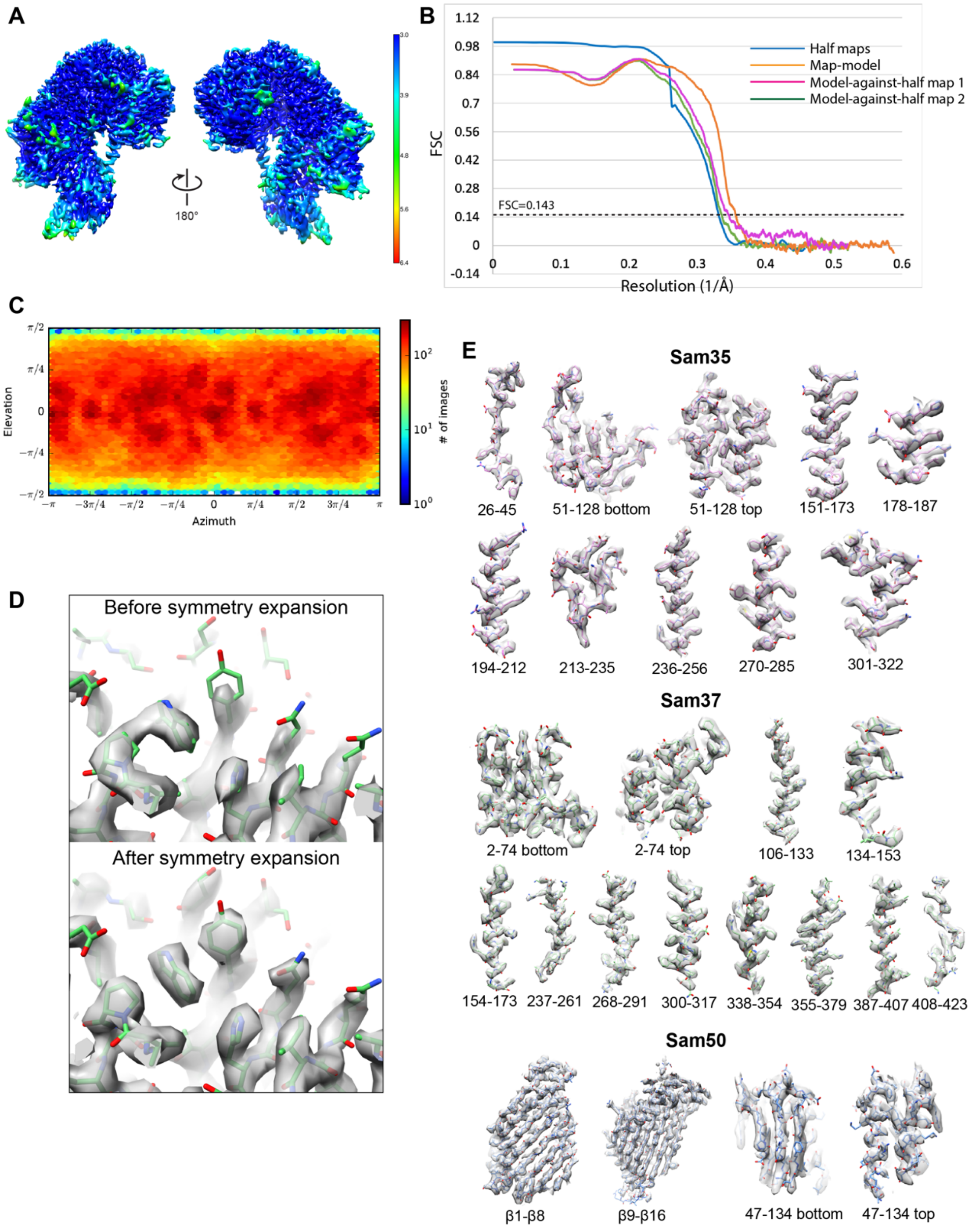

Supplementary Figure 6. Resolution estimation of cryoEM single particle reconstruction of the SAM complex in detergent GDN with symmetry expansion. A. Local resolution map calculated by cryoSPARC2**. B.** FSC curves. **C.** Orientation distribution plot. **D.** The resolution and quality of the cryoEM density map were both improved after symmetry expansion as demonstrated in a representative region in Sam37. The density maps (grey) are displayed at 1.0 σ (calculated locally). **E.** Superposition of the cryoEM densities and atomic model of the SAM complex in detergent GDN.

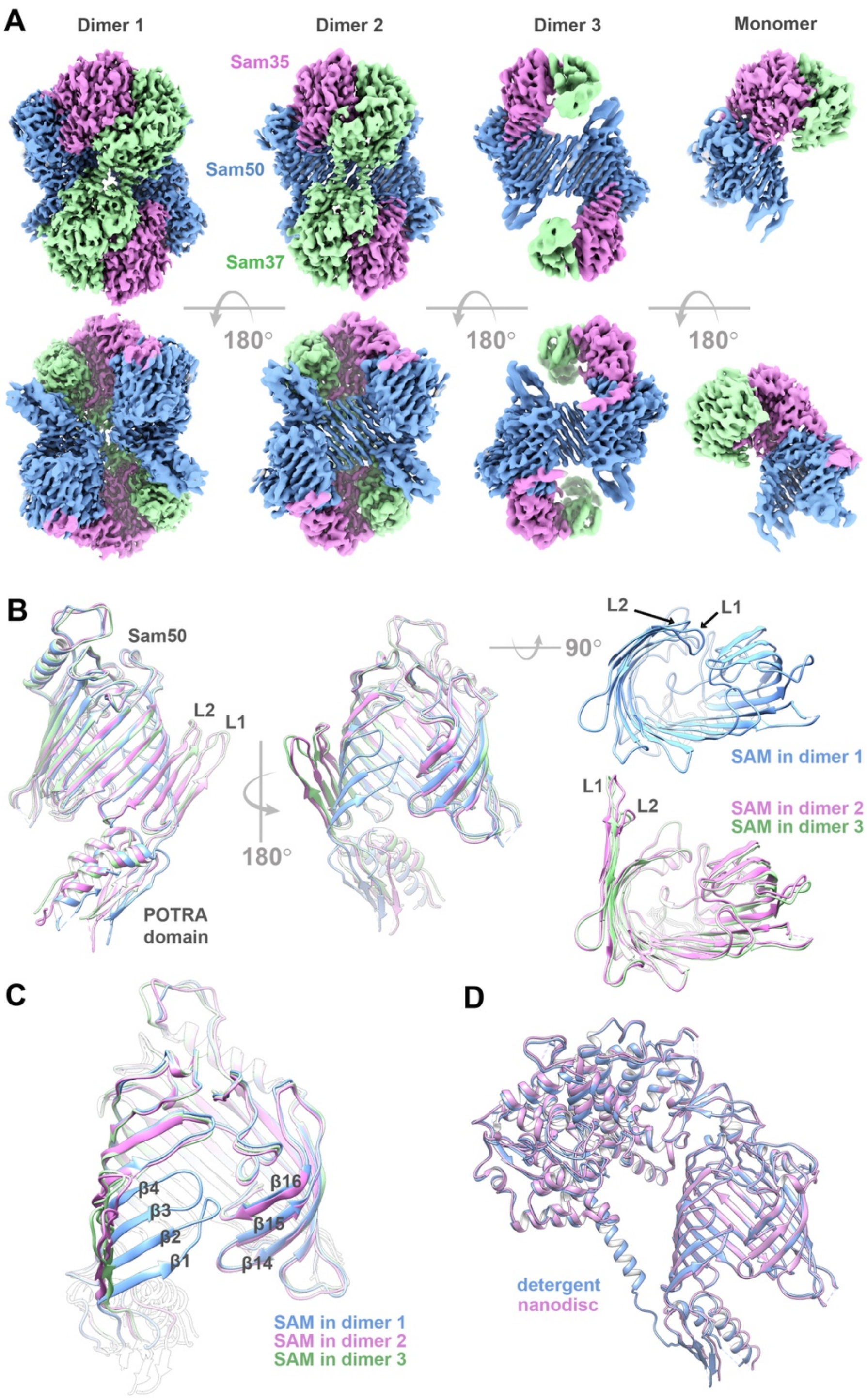

Supplementary Figure 7. Conformations of Sam50 in detergent GDN and in lipid nanodiscs. A. Comparison of the cryoEM density maps of the SAM complex as dimers and monomer in GDN. **B.** Superposition of Sam50 from dimer 1 (blue), dimer 2 (orchid), and dimer 3 (green). Dimer 2 and dimer 3 exhibit a barrel opened by β1-β4. **C.** Comparison of the lateral gate of Sam50 in each dimer. **D.** Comparison of the similar structures of the SAM complex in GDN (blue) and lipid nanodiscs (orchid).

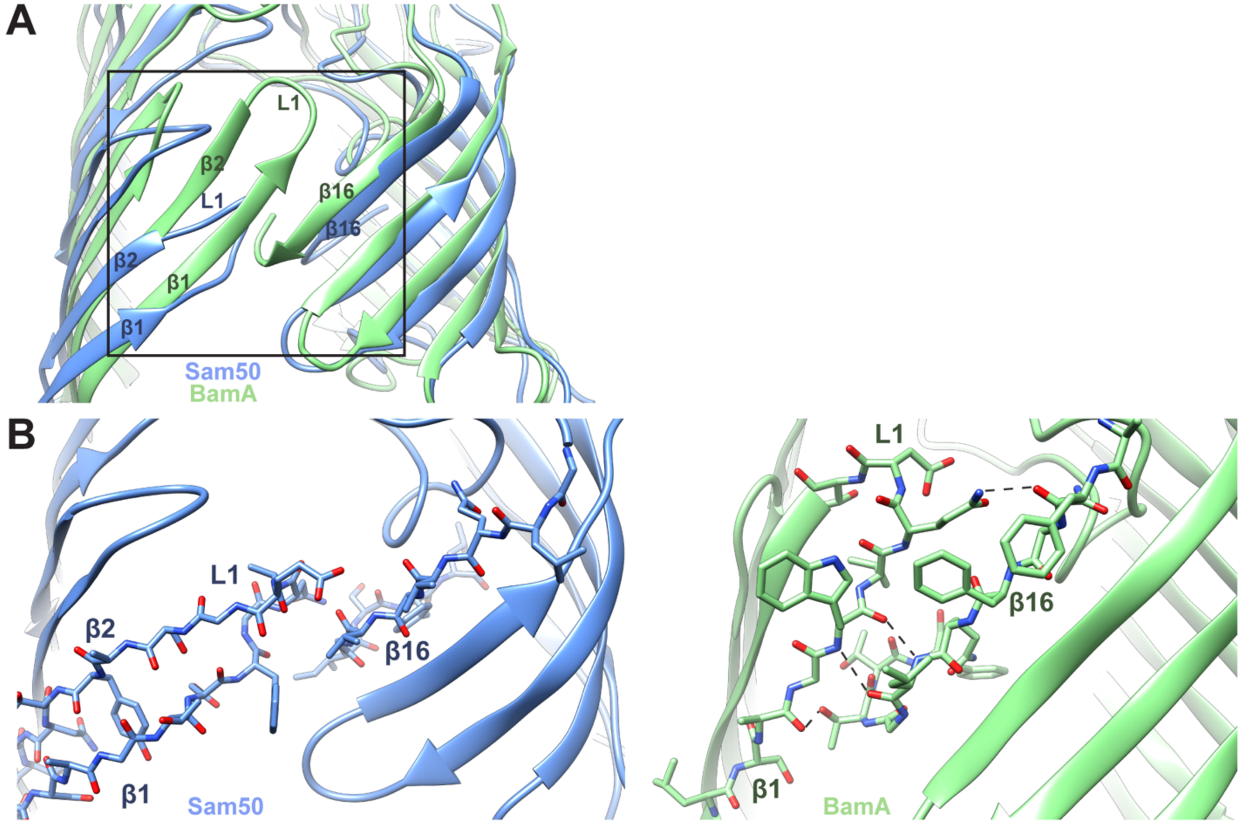

Supplementary Figure 8. Interactions between β1 and 16 in Sam50 and BamA. A. Comparison of the lateral gate in Sam50 (blue) and BamA (green)**.** Strands β1-β2, β16 and loop 1 are indicated. The boxed area is zoomed in for each protein in [B]. **B.** Lateral gate of Sam50 on the left, and *Neisseria* BamA (PDB:4K3B) on the right. Hydrogen bonds are shown as dashed lines.

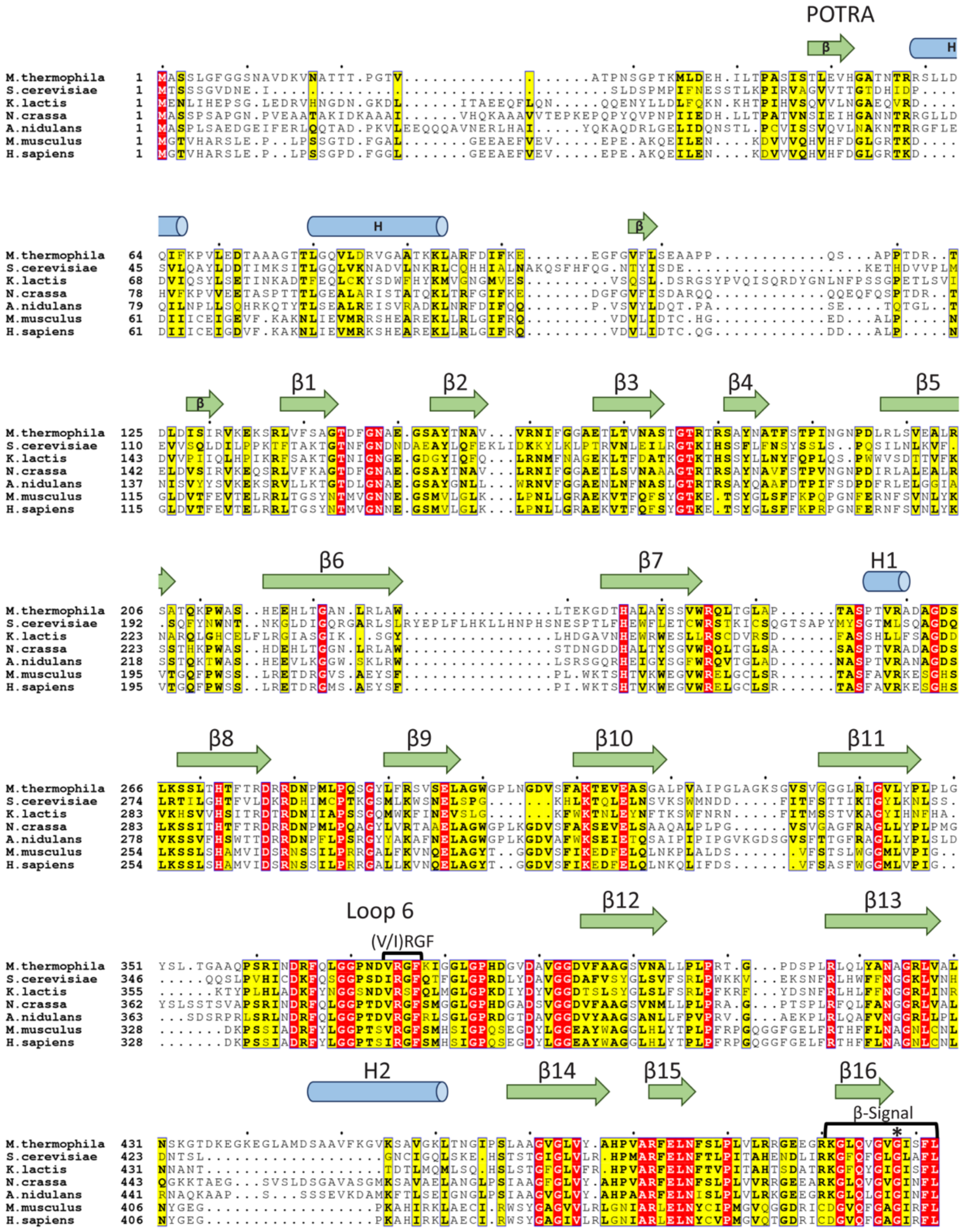

Supplementary Figure 9. Structure-coded sequence alignment for Sam50. Secondary structure assigned based on high-resolution structure of *M. thermophila* SAM complex in detergent. Blue cylinders represent α-helices, green arrows represent β-strands. Asterisk identifies glycine that forms a kink in β16. *M.thermophila* (*Myceliophthora thermophila,* Uniprot: G2QFF9), *S.cerevisiae* (*Saccharomyces cerevisiae,* Uniprot: P53969), *K.lactis* (*Kluyveromyces lactis,* Uniprot: Q6CNZ6), *N.crassa* (*Neurospora crassa,* Uniprot: V5IKW7), *A.nidulans* (*Aspergillus nidulans,* Uniprot: C8VCB1), *M.musculus* (*Mus musculus,* Uniprot: Q8BGH2), *H.sapiens* (*Homo sapiens,* Uniprot: Q9Y512)

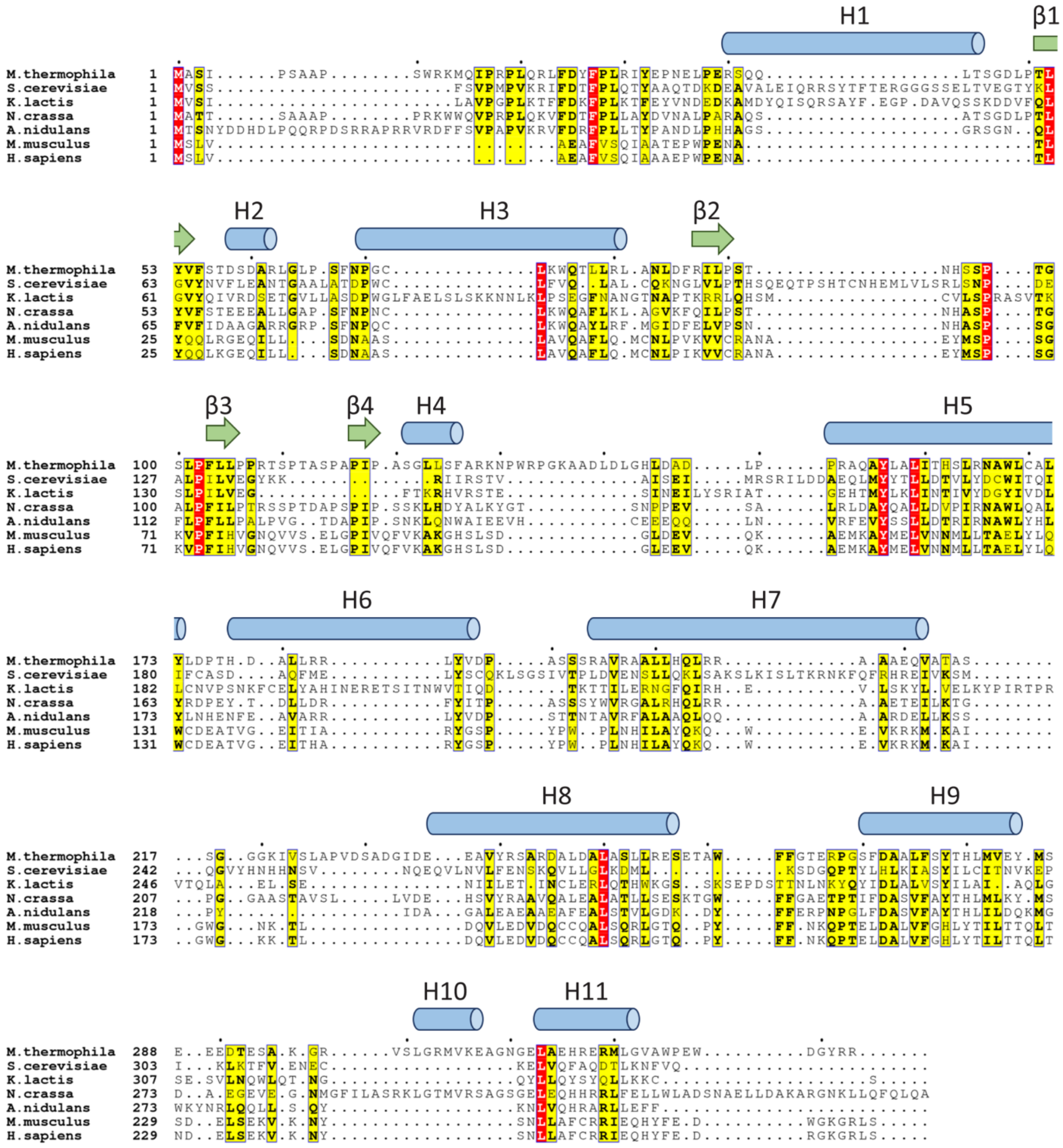

Supplementary Figure 10. Structure-coded sequence alignment for Sam35. Secondary structure assigned based on high-resolution structure of *M. thermophila* SAM complex in detergent. Blue cylinders represent α-helices, green arrows represent β-strands. *M.thermophila* (*Myceliophthora thermophila,* Uniprot: G2QAT9), *S.cerevisiae* (*Saccharomyces cerevisiae,* Uniprot: P14693), *K.lactis* (*Kluyveromyces lactis,* Uniprot: Q6CMW8), *N.crassa* (*Neurospora crassa,* Uniprot: V5IPB4), *A.nidulans* (*Aspergillus nidulans,* Uniprot: Q5BFM3), *M.musculus* (*Mus musculus,* Uniprot: O88441), *H.sapiens* (*Homo sapiens,* Uniprot: O75431).

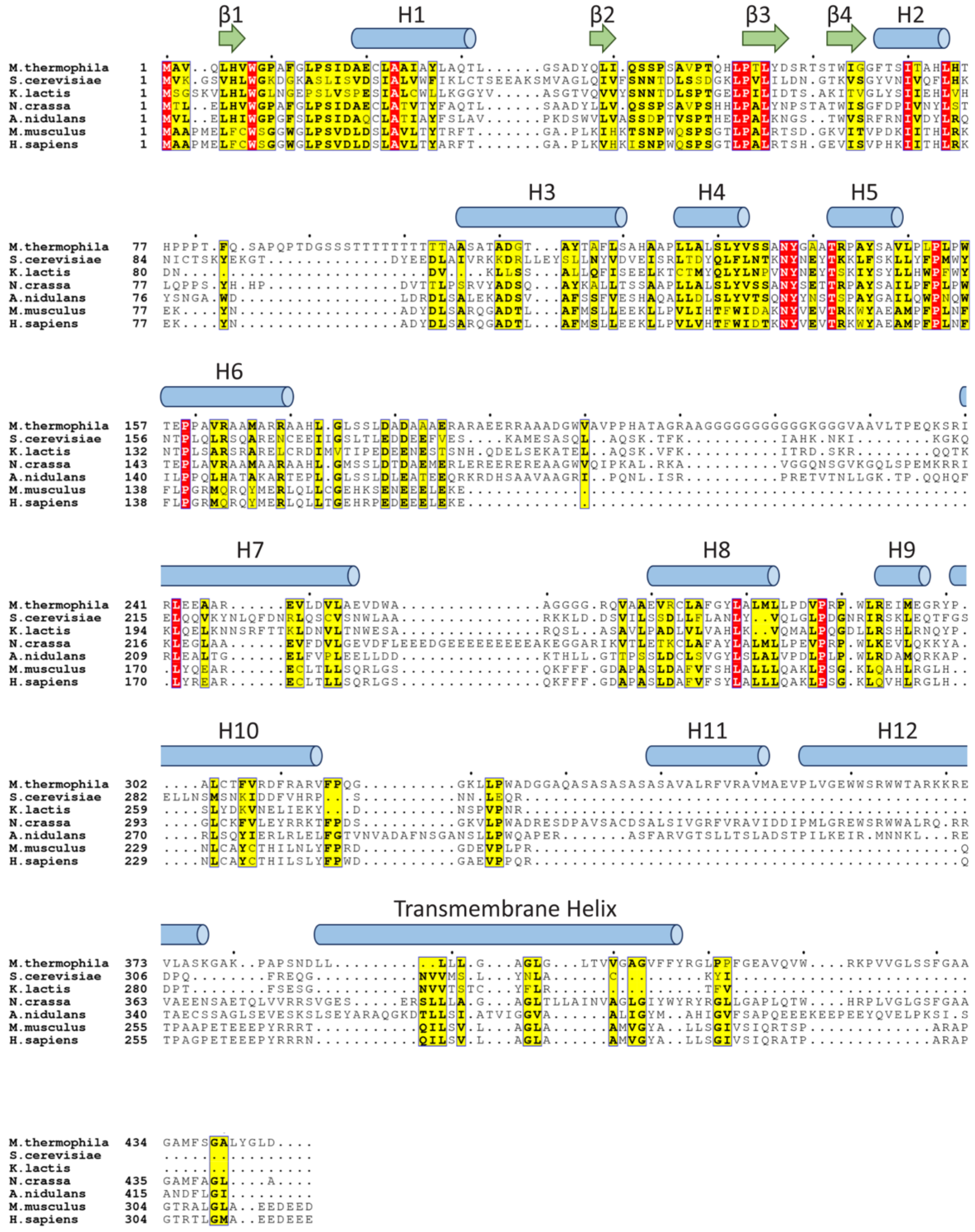

Supplementary Figure 11. Structure-coded sequence alignment for Sam37. Secondary structure assigned based on high-resolution structure of *M. thermophila* SAM complex in detergent. Blue cylinders represent α-helices, green arrows represent β-strands. *M.thermophila* (*Myceliophthora thermophila,* Uniprot: G2Q6R7), *S.cerevisiae* (*Saccharomyces cerevisiae,* Uniprot: P50110), *K.lactis* (*Kluyveromyces lactis,* Uniprot: Q6CII7), *N.crassa* (*Neurospora crassa,* Uniprot: Q7SFC4), *A.nidulans* (*Aspergillus nidulans,* Uniprot: Q5B6M7), *M.musculus* (*Mus musculus,* Uniprot: P47802), *H.sapiens* (*Homo sapiens,* Uniprot: Q13505-3).

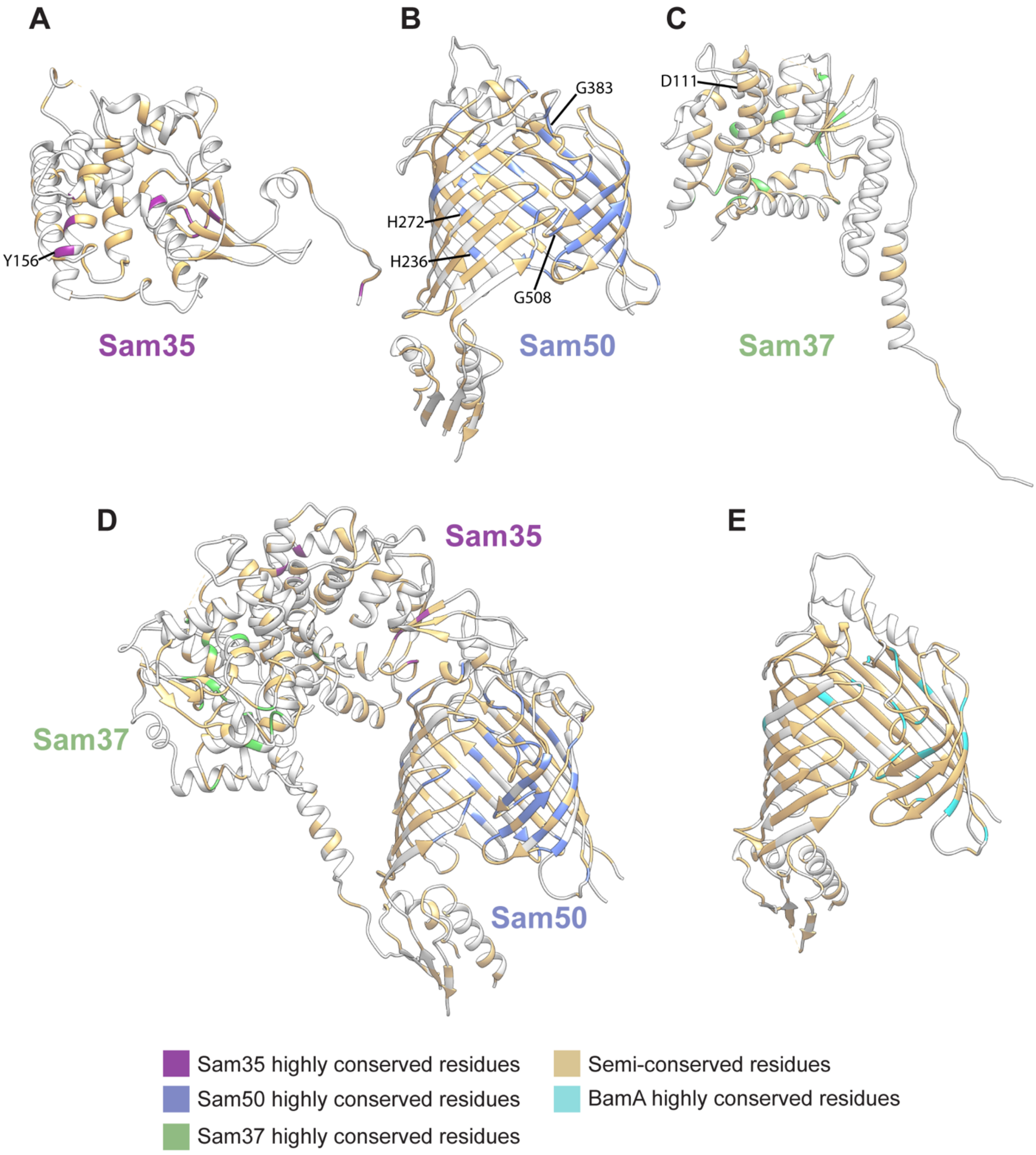

Supplementary Figure 12. Highly conserved residues in each subunit of the SAM complex. **A.** Sam35. **B.** Sam50. **C.** Sam37. **D.** The entire SAM complex. **E.** Sam50 residue conservation with *Neisseria gonorrhoeae* BamA. Highly conserved residues are in colors specific for each subunit and semi-conserved residues are in gold for all subunits.

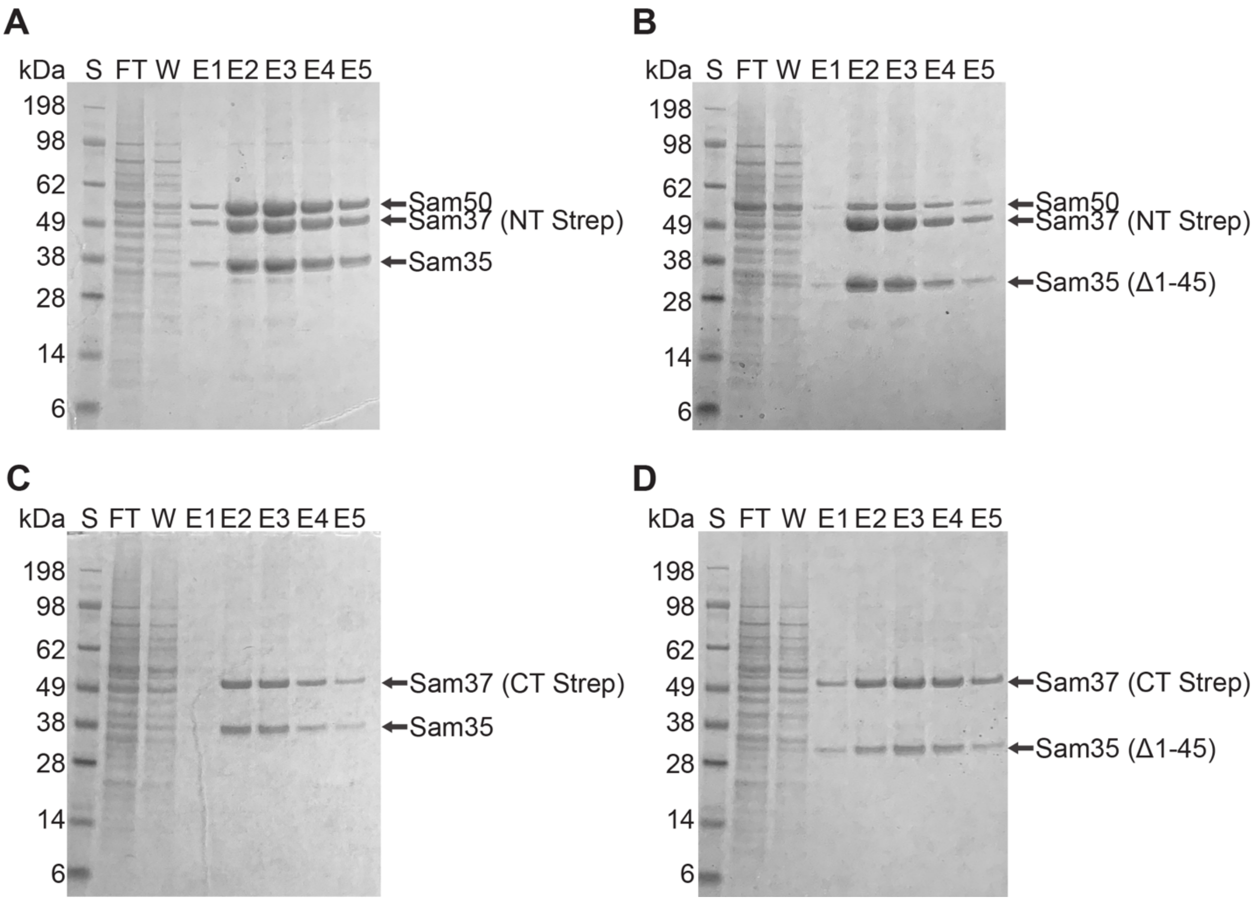

Supplementary Figure 13. SAM complex assembly with Sam35 N-terminal truncation (Δ1-45). Solubilization and strep affinity purification in LMNG. Assays conducted in parallel, starting with equal concentrations of mitochondria. SDS PAGE of strep affinity purification fractions. **A.** Full length ternary complex. **B.** Ternary complex with Sam35 N-terminal truncation. **C.** Full length Sam35 + Sam37 complex. **D.** Sam35 N-terminal truncation + Sam37 complex. The experiments in **A** and **C** were repeated at least three separate times with similar results. The experiments in **B** and **D** were conducted once in parallel, along with full length constructs (**A** and **C**). Source data for A-D are provided as a Source Data file.
S= Standard, FT= Flow through, W= Wash, E= Elution fractions. 50% load FT, W and E undiluted.

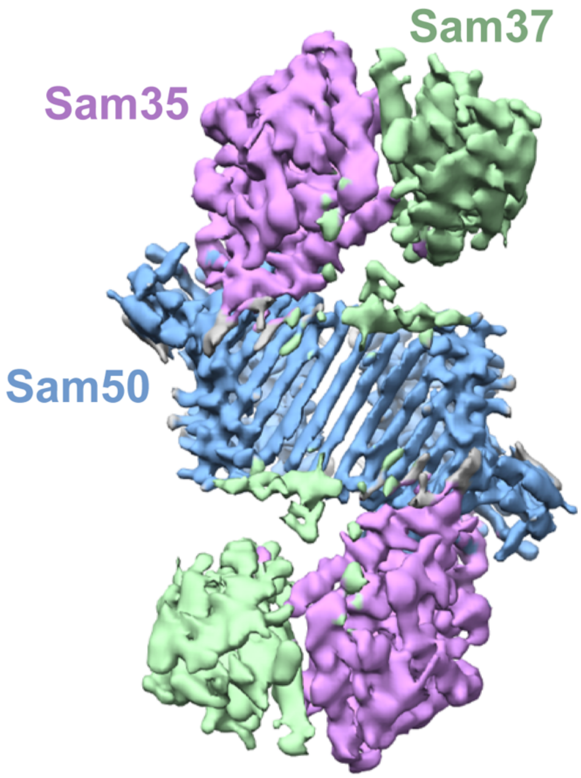

Supplementary Figure 14. 4.1-Å resolution cryoEM density map of SAM complex containing Sam50 POTRA deletion (Δ1-135) in detergent GDN. Deletion of the entire POTRA domain (residues 1-135) of Sam50 did not alter the assembly of the Sam50-Sam35-Sam37 heterotrimeric complex.

Protein	Mt Sam35		Mt Sam50		Mt Sam37	
	Sam35	Sam50	Sam50	Sam37	Sam37	Sam35
**Nr of atoms**						
in interface	183 (8.0%)	219 (6.6%)	113 (3.4%)	98 (3.7%)	213 (8.1%)	209 (9.1%)
on the surface	1433 (62.4%)		2089 (63.4%)		1632 (62.1%)	
Total	2295 (100.0%)		3295 (100.0%)		2628 (100.0%)	
**Buried Surface area (A^2)**	1,972.8 (12.0%)	1,865.4 (8.0%)	924.4 (4.0%)	966.9 (5.0%)	2,011.6 (10.4%)	1945.8 (11.8%)
**Accessible Surface area (A^2)**	16,466.6		23,272.2		19,312.1	
**Solvation Energy (kcal/mol)**	-19.9		-7.9		-14.3	
Salt bridges in interface	3		0		2	
H-bonds in interface	15		8		9	
**Volume (A^3)**	36,180		52,820		41,670	
**Buried Surface area in cplx (A^2)**	3,918.6		2,789.8		2,978.5	
**Total surface area of cplx (A^2)**	49,920					
**Total buried area in cplx (A^2)**	9,686.9					

Supplementary Table 1. QT-Pisa analysis of molecular interfaces in the high-resolution detergent SAM complex. Volume value from Chimera analysis, number of salt bridges and hydrogen bonds in interface from PyMOL analysis.

Hydrogen bonds				Salt bridges		
**Sam37**	**distance**	**Sam35**		**Sam37**	**distance**	**Sam35**
H-28 N	3.3	L174 O		D111 OD1	3.3	R152 NE
A-31 O	3.1	A231 N		R363 NH2	2.4	D229 OD2
T49 O	2.4	K221 NZ				
Q50 O	3.1	I222 N				
S69 OG	3.4	R241 NH2				
D111 OD1	3.1	Y156 OH				
R168 NH1	3.4	L225 O				
E353 OE2	3.5	L225 N				
K370 NZ	2.4	I234 O				
**Sam37**	**distance**	**Sam50**		**Sam37**	**distance**	**Sam50**
F406 O	2.5	R137 NH2				
F413 N	3.2	T55 O				
G414 N	2.6	T55 O				
A416 O	3.4	G53 N				
Q418 O	3.5	V51 N				
Q418 NE2	3.3	A54 O				
V419 O	3.0	R59 NH1				
V419 O	3.4	R59 NH2				
**Sam50**	**distance**	**Sam35**		**Sam50**	**distance**	**Sam35**
R259 NH1	2.6	S95 O		D386 OD1	2.9	R108 NH1
A260 O	3.3	R40 NH2		D386 OD2	2.8	R108 NH2
A262 O	2.7	T92 OG1		K433 NZ	3.4	E36 OE2
G383 O	3.3	N35 ND2				
G387 N	3.0	T112 O				
L430 O	3.1	E39 N				
N431 ND2	2.7	E36 OE1				
S432 N	2.7	E39 OE1				
S432 OG	2.9	E39 OE2				
L491 O	2.7	L29 N				
L491 O	3.5	R30 N				
L493 O	3.2	Y32 N				
L493 N	2.8	R30 O				
R495 N	3.1	Y32 O				
E497 OE2	3.3	N35 ND2				

Supplementary Table 2. List of interfacing residues in the SAM complex, identified by QT Pisa and PyMOL.

Yellow indicates highly conserved residues (≥60%), red indicates absolutely conserved residues (across 7 species) from sequence alignments. Italics indicates residue conserved across 7 species of Sam50 and *N. gonorrhoeae* BamA.

Sam35	Sam37	Sam50		
M1*	M1*	M1*	K311	G425
F27	W8	T144	E315	L427
L52	A23	G147	G342	G471
L73	L52	N148	D364	G473
P97	P53	G176	F366	V475
P102	L55	T177	G369	A481
Y156	I70	G221	G370	R482
L159	L74	H236	R375	E484
L250	N137	R245	F377	L485
L313	Y138	S255	G383	N486
	T142	G263	P384	P490
	P153	H272	D389	G502
	P159	D277	G392	Q504
	L242	R279	G393	G506
	L279	P285	P408	G508
	P288	G288	R417	F511
		E296	N423	L512

Supplementary Table 3. Conserved residues determined from structure-based sequence alignments.

Asterisk identifies residues that are not visible in the structure. Residues identified are absolutely conserved across 7 species, identified by sequence alignments.

M. thermophila Sam50			*N. gonorrhoeae* BamA		
Residue 1	Residue 2	Distance (Å)	Residue 1	Residue 2	Distance (Å)
No Interactions Identified			A430 O β1	T790 OG1 β16	3.0
			W432 N β1	L788 O β16	3.1
			W432 O β1	L788 N β16	3.0
			Q434 NE2 L1	F784 O β16	3.0

Absolutely conserved Sam50 or BamA residue

Highly conserved residue (≥60% for Sam50, ≥80% for BamA)

Conserved across 7 species Sam50 and N. gonorrhoeae BamA

Supplementary Table 4. Lateral gate interactions in Sam50 and BamA. Lateral gate interactions in Sam50 detergent structure and BamA (PDB:4K3B), identified using PyMOL.

Application	Cloning Method	Product	Primer 1	Primer 2
Expression Vector Assembly	Golden Gate	pBEVY-GL	CACACCAGGTCTCAAATACCGAGCTCGAATTCGACACT	CACACCAGGTCTCAGCAGACATTTTTTTTTTCTCCTTGACGTTAAAGTATAGAGGT
	Golden Gate	pBEVY-GT	CACACCAGGTCTCAAATACCGAGCTCGAATTCGACACT	CACACCAGGTCTCATGGTGGTGATGGTGATGGTGGTGATGAGACATTTTTTTTTTCTCCTTGACGTTAAAGTATAGAGGT
	Infusion	pBEVY-GU	TACCGAGCTCGAATTCGACACTTC	TTTTTTCTCCTTGACGTTAAAGTATAGAGGTATATTAAC
	Golden Gate	Sam50 NT His with TEV	CACACCAGGTCTCAACCATCACGGTTCAGAAAACTTGTATTTCC	CACACCAGGTCTCATATTATTACAAAAATGAAATACCTACACCGACTTGC
	Golden Gate	Sam35 no tag	CACACCAGGTCTCACTGCTTCAATTCCATCTGCTGCACC	CACACCAGGTCTCATATTATTATCTTCTGTAACCATCCCATTCTGGCC
	Infusion	Sam37 CT Twin-Strep with TEV	AATTCGAGCTCGGTATTATTACTTCTCAAATTGTGGATGGGACCA	GTCAAGGAGAAAAAAAAAATGTCTGCTGTTCAATTGCATGTTTG
	Infusion	Sam37 NT Twin-Strep with TEV	AATTCGAGCTCGGTATTATTAATCCAAACCGTATAAAGCACCAGAAAAC	GTCAAGGAGAAAAAAAAAATGTCTAGCGCTTGGAGCCAC
Mutagenesis	Q5 Site-Directed Mutagenesis	Sam37 NT Twin-Strep no TEV	GGTGGGGCTGTTCAATTG	CTTCTCAAATTGTGGATGGG
		Sam50 no tag	GCCTCCTCCTTAGGTTTC	AGACATTTTTTTTTTCTCCTTGAC
		Sam50 POTRA deletion (Δ135)	TCCAGATTAGTTTTCTCTGCTG	AGACATTTTTTTTTTCTCCTTGAC
		Sam35 N-terminal truncation (Δ45)	TCTGGTGACTTGCCAACA	CATTTTTTTTTTCTCCTTGACGTTAAAG

Supplementary Table 5. List of primers.
