# Supplementary material for: Structural insight into mitochondrial β-barrel outer membrane protein biogenesis
Source: Nat Commun. 2020 Jul 3;11:3290. doi: 10.1038/s41467-020-17144-1 (PMC7335169; doi:10.1038/s41467-020-17144-1)
Supplement: Supplementary file 3 — Description of Additional Supplementary Information [file 41467_2020_17144_MOESM3_ESM.pdf]

Description of Additional Supplementary Files

File Name: Supplementary Movie 1

Description: Conformational changes in the lateral gate of Sam50. Sam50 (blue) morphing between the partially closed conformation (dimer 1) and the open conformation (dimer 3). β1-β4 rotate outward to open the lateral gate. These conformational changes illustrate the flexibility within the Sam50 β-barrel to accommodate a substrate as it folds. Sam35 (orchid), Sam37 (light green) and the cryoEM densities of the SAM complex in nanodiscs (light yellow) are also shown. The nanodisc clearly outlines the membrane for the complex.
