# Supplementary material for: Structural insight into mitochondrial β-barrel outer membrane protein biogenesis
Source: Nat Commun. 2020 Jul 3;11:3290. doi: 10.1038/s41467-020-17144-1 (PMC7335169; doi:10.1038/s41467-020-17144-1)
Supplement: Supplementary file 5 — Reporting Summary [file 41467_2020_17144_MOESM5_ESM.pdf]

nature research

Corresponding author(s): Jiansen Jiang
Susan K. Buchanan

Last updated by author(s): May 30, 2020

# Reporting Summary

Nature Research wishes to improve the reproducibility of the work that we publish. This form provides structure for consistency and transparency in reporting. For further information on Nature Research policies, see our Editorial Policies and the Editorial Policy Checklist.

## Statistics

For all statistical analyses, confirm that the following items are present in the figure legend, table legend, main text, or Methods section.

| n/a | Confirmed | |
|---|---|---|
| ☒ | ☐ | The exact sample size (*n*) for each experimental group/condition, given as a discrete number and unit of measurement |
| ☒ | ☐ | A statement on whether measurements were taken from distinct samples or whether the same sample was measured repeatedly |
| ☒ | ☐ | The statistical test(s) used AND whether they are one- or two-sided<br>*Only common tests should be described solely by name; describe more complex techniques in the Methods section.* |
| ☒ | ☐ | A description of all covariates tested |
| ☒ | ☐ | A description of any assumptions or corrections, such as tests of normality and adjustment for multiple comparisons |
| ☒ | ☐ | A full description of the statistical parameters including central tendency (e.g. means) or other basic estimates (e.g. regression coefficient) AND variation (e.g. standard deviation) or associated estimates of uncertainty (e.g. confidence intervals) |
| ☒ | ☐ | For null hypothesis testing, the test statistic (e.g. *F*, *t*, *r*) with confidence intervals, effect sizes, degrees of freedom and *P* value noted<br>*Give P values as exact values whenever suitable.* |
| ☒ | ☐ | For Bayesian analysis, information on the choice of priors and Markov chain Monte Carlo settings |
| ☒ | ☐ | For hierarchical and complex designs, identification of the appropriate level for tests and full reporting of outcomes |
| ☒ | ☐ | Estimates of effect sizes (e.g. Cohen's *d*, Pearson's *r*), indicating how they were calculated |

*Our web collection on statistics for biologists contains articles on many of the points above.*

## Software and code

Policy information about availability of computer code

| | |
|---|---|
| Data collection | Leginon (version 3.3) |
| Data analysis | MotionCor2 (version 1.2.1), CTFFIND4 (version 4.1.10), RELION3 (version 3.0.7), cryoSPARC2 (version 2.9.0), Gautomatch (version 0.56), COOT (version 0.8.5), PHENIX (version 1.15), ITASSER (version 5.1), Phyre2 (version 2.0), Rosetta 3 (2019), UCSF Chimera (version 1.13.1), UCSF ChimeraX (version 0.91), QT Pisa (version 2.1.0), PyMOL (version 2.3), T-Coffee Expresso (version 13.41.0.28bdc39), Jalview (version 2.10.2b2), ESPript 3.0 (version 3.0.7) |

For manuscripts utilizing custom algorithms or software that are central to the research but not yet described in published literature, software must be made available to editors and reviewers. We strongly encourage code deposition in a community repository (e.g. GitHub). See the Nature Research guidelines for submitting code & software for further information.

## Data

Policy information about availability of data

All manuscripts must include a data availability statement. This statement should provide the following information, where applicable:

- Accession codes, unique identifiers, or web links for publicly available datasets
- A list of figures that have associated raw data
- A description of any restrictions on data availability

Atomic coordinates and structure factors for SAM complex structures have been deposited in the EMDB and wwPDB under accession codes EMD-21913/6WUH, EMD-21914/6WUJ, EMD-21915/6WUL, EMD-21916/6WUM, EMD-21917/6WUN, and EMD-21918/6WUT. Myceliophthora thermophila SAM complex sequences are available from uniprot.org under UniProt IDs G2QAT9 (Sam35), G2Q6R7 (Sam37), and G2QFF9 (Sam50). The source data underlying Supplementary Figures 2 and 13 are provided as a Source Data file. Source data for all figures and files is available from the authors upon request.

# Field-specific reporting

Please select the one below that is the best fit for your research. If you are not sure, read the appropriate sections before making your selection.

- [x] Life sciences
- [ ] Behavioural & social sciences
- [ ] Ecological, evolutionary & environmental sciences

For a reference copy of the document with all sections, see nature.com/documents/nr-reporting-summary-flat.pdf

# Life sciences study design

All studies must disclose on these points even when the disclosure is negative.

| | |
|---|---|
| Sample size | Sample size was not predetermined. Microscope time was the limiting factor for cryo-EM data collection. Data was collected until the density resolution was sufficient for accurate model building. |
| Data exclusions | Cryo-EM data processing results in the exclusion of poor quality particles. Processing protocols using RELION3 and cryoSPARC programs were followed, as is standard in the field. |
| Replication | Purification of the SAM complex in detergent (LMNG and GDN) and lipid nanodiscs was repeated at least three times with similar results (Supplementary Figure 2). Pull down assay of full length ternary SAM complex and Sam35+Sam37 complex was completed at least three separate times with similar results (Supplementary Figure 13). Pull down assay of Sam35 truncation constructs (ternary complex and Sam35 +Sam37 complex) was completed once, in parallel with full length constructs (Supplementary Figure 13). For single particle analysis by cryo-EM, 2D classification was performed 7 times during data processing and these 2D class averages were observed in all 2D classification results for nanodiscs (supp fig 3b) and 3 times for detergent (supp fig 5). |
| Randomization | Randomization was not used, and it is not applicable to this study. |
| Blinding | Blinding was not used, and it is not applicable to this study. |

# Reporting for specific materials, systems and methods

We require information from authors about some types of materials, experimental systems and methods used in many studies. Here, indicate whether each material, system or method listed is relevant to your study. If you are not sure if a list item applies to your research, read the appropriate section before selecting a response.

## Materials & experimental systems

| n/a | Involved in the study | |
|---|---|---|
| x | | Antibodies |
| | x | Eukaryotic cell lines |
| x | | Palaeontology and archaeology |
| x | | Animals and other organisms |
| x | | Human research participants |
| x | | Clinical data |
| x | | Dual use research of concern |

## Methods

| n/a | Involved in the study | |
|---|---|---|
| x | | ChIP-seq |
| x | | Flow cytometry |
| x | | MRI-based neuroimaging |

## Eukaryotic cell lines

Policy information about cell lines

| | |
|---|---|
| Cell line source(s) | S. cerevisiae W303.1B (MATa {leu2-3,112 trpl-1 canl-100 ura3-1 ade2-1 his3-11,15}) |
| Authentication | Cell line was used for heterologous protein expression. No authentication performed, but for the use of selection markers. |
| Mycoplasma contamination | N/A |
| Commonly misidentified lines (See ICLAC register) | *Name any commonly misidentified cell lines used in the study and provide a rationale for their use.* |
